# Detection of antifungal drug-resistant and *ERG11* gene mutations among clinical isolates of *Candida* species isolated from Khartoum, Sudan.

**DOI:** 10.12688/f1000research.24854.2

**Published:** 2021-01-29

**Authors:** Ahmed Osman Mohamed, Malik Suliman Mohamed, Mohamed Abdelrahman Hussain, Ibrahim Fatahalrahman Ahmed

**Affiliations:** 1 Department of Pharmaceutical Microbiology, Faculty of Pharmacy, International University of Africa, Khartoum, 11111, Sudan; 2 Department of Pharmaceutics, Faculty of Pharmacy, Sudan International University, Khartoum, 11111, Sudan; 3 Department of Pharmaceutics, College of Pharmacy, Jouf University, Sakaka, Al Jouf, P.O.Box 2014, Saudi Arabia; 4 Department of Pharmaceutics, Faculty of Pharmacy, University of Khartoum, Khartoum, P. O. Box 1996, Sudan; 5 Department of Microbiology, Faculty of Pure and Applied Science, International University of Africa, Khartoum, 11111, Sudan

**Keywords:** Candida species, fluconazole resistance, ERG11

## Abstract

**Background:** *Candida* species are one of the most important opportunistic fungal pathogens that cause both superficial and systemic infections, especially in immunocompromised individuals. Considering the sharp increase in the rate of *Candida* infections, and resistance to commonly used antifungal agents in the last decades; this study was conducted to determine the rate of resistance among clinical isolates of *Candida* species, and to characterize some of the resistant genes among resistant isolates collected in Khartoum.

**Methods:** This is a cross-sectional laboratory-based study included 100 pre-screened *Candida* species isolates from Khartoum state hospitals. Chromogenic media was used for *Candida* isolation and/or identification. The standard disc diffusion method was performed to investigate the susceptibility to fluconazole, itraconazole, and amphotericin. Following genomic DNA extraction, the entire *ERG11* gene was amplified from some *C. albicans* resistant isolates, sequenced, and further analyzed.

**Results:** Out of 100 clinical isolates collected, 51% were *C. albicans*, followed by *C. glabrata* (31%), *C. krusie* (8%), *C. tropicals* (5%), and *C. dupliniens* (5%). Resistance rate was 23% for fluconazole, 4% for itraconazole, while there were no amphotericin resistant isolates detected. *C. albicans* *ERG11* gene sequence reveals 15 different mutations. Among these, three (D116E, E266D, and V488I) were missense mutations; however, these substitutions do not contribute to fluconazole resistance.

**Conclusion:** *C* *. albicans* was found to be the most common species. Resistance against fluconazole was observed most frequently; however, mutations in *ERG11* are unlikely to be the reason behind fluconazole resistance among these isolates.

## Introduction

The genus *Candida* is a dimorphic opportunistic fungal pathogen that colonizes the vagina, gastrointestinal and mucosal oral cavity of immunocompetent individuals. In contrast, critically ill and/or immunocompromised patients frequently develop *Candida* infection that range from superficial to systemic infections ^1^. *Candida* comprises over 150 species, of which 17 are prevalent and known to infect humans; these include *Candida albicans, Candida glabrata, Candida parapsilosis, Candida tropicalis, *and *Candida krusei* ^2, 3^.

Since the 1980s, there has been a steady increase in the incidence and prevalence of serious secondary systemic fungal infections ^4^. The risk factors for developing systemic or superficial *Candida* infections include intensive care unit admissions, HIV infection, organ transplantation, cancer and anticancer drugs, diabetes mellitus and other demographic factors such as age and sex ^5– 9^.

Various antifungals are used for treatment of *Candida* infections, among them, azoles which showed good activity and are relatively safe, however, resistance to this group is occurring more frequently ^10^. On the other hand, resistance of *Candida* species against other antifungals such as Polyenes, Echinocandins, and Allylamines has not been reported extensively ^11^. 

There are different molecular mechanisms through which eukaryotic cells may develop drug resistance such as alteration in efflux pump, alteration in intracellular drug processing i.e. modification or degradation, alterations in the target enzyme and/ or other enzymes. Among these mechanisms, alteration of the targeted enzyme and alteration of efflux pump are the most common in *Candida* species ^12^.

### Alteration in the target enzyme

The azoles bind to and inhibit the activity of 14 α-demethylase, a key enzyme in the fungal ergosterol synthesis pathway, which belongs to the cytochrome P450 family ^10^. One of the known potential resistance mechanism of azoles is alteration in the ergosterol syntheses pathway. *Candida* species can develop resistance by mutation/s in the gene ( *ERG11*) which codes for the enzyme 14α-demethylase ^13^ *.* The point mutation in the *ERG11* gene can result in an amino acid substitution which in turn produces a conformational change in the enzyme and decreases the affinity to azoles, however, susceptibilities are not affected equally by different mutations as the presence of some mutations such as Y132H, R467K, and I471T confirm resistance, on the other hand, mutations such as E266D does not affect the resistance ^14^. In addition, drug resistance might develop through overexpression of the *ERG11* gene through increased mRNA level which might increase the concentration of the enzyme in comparison to sensitive isolates ^15^.

### Efflux pump

Efflux pumps are the basic mechanism in most eukaryotic cells by which unwanted toxic materials are forced out of the cell. Two types of efflux pumps have been identified: ATP binding cassette (ABC) transporters and major facilitator superfamily (MFS) ^16^. ABC transporters are pumps that are actively associated with the efflux of potentially toxic molecules to the cell, and they are primarily hydrophobic and lipophilic ^17^. Many different pumps are known to belong to the different families, such as *Candida* resistance ( *CDR1&2*) genes that are related to the PDR5 family, and known to be associated with resistance to antifungals ^15^. The MFS pumps work by antiproton power i.e. proton pumped inside the cell and hydrophobic and/or lipophilic material pumped outside the cell. This is coded by multidrug resistance gene (MDR1) which found to be overexpressed in fluconazole resistant isolates, however, the gene was not overexpressed in other azoles, ketoconazole and itraconazole, resistant strains ^18^. Some authors have tried to link overexpression of CDR1, 2 and MDR1, concluding that deletions of these genes will result in more susceptible isolate than each gene alone ^12^.

Among different antifungals, Fluconazole was the most commonly prescribed, amphotericin and itraconazole remains to be the cornerstone in the management of systemic fungal infections in Sudan since echinocandins are not yet registered (Sudan National Essential Medicine List- 2014) ^19^. Due to the scarcity of reports about the rate of drug resistance and resistant genes among *Candida* species in the study area, this study was conducted to screen the susceptibility of different *Candida* species towards commonly used antifungals, and to identify the role of *ERG11* gene mutation/s in the development of fluconazole resistance. To this end, we collected and identified isolates of *Candida* species, selected the most resistant isolates, amplified and sequenced the conserved domain of the *ERG11* gene and detected the impact of the mutation(s) on the enzyme 14α-demethylase’s structure and function using *in silico* tools ^20, 21^.

## Methods

### Ethical statement

This study was reviewed and approved by the Research ethical committee, Faculty of Medicine & Health Science, International University of Africa (IUA) (6-2017). Oral informed consent was obtained from the participating patients, when their hospital laboratory result was positive for *Candida* species. Oral consent was obtained over written consent (where it was recorded), since the majority of the patients included in this study were illiterate (in case of minors, consent was obtained from parents or guardians). The structure of the consent was approved by the Research Ethics Committee.

### Study design, area and participants

This was a cross-sectional laboratory-based study using clinical isolates of *Candida* species. Clinical isolates were collected from different Khartoum state hospitals in a period between September 2017 to September 2018, all clinical isolates primarily identified as *Candida* species regardless of age, gender, and site of isolations, were included. Samples and demographic data were obtained directly from the patients within each hospital after consent was obtained by the principle investigators.

### Sample size calculation

Sample size was calculated using the following formula on cross-sectional studies ^22^:

n = Z ^2^ * P (1-P) / d ^2^

Where, n = desired sample size, Z = critical value and a standard value for the corresponding level of confidence (1.96), P = expected frequency of resistance obtained from previous studies (7%) ^23, 24^, d = margin of error (0.05).

n = 1.96 ^2 ^* 0.07 (1-0.07) / (0.05) ^2 ^= 100 samples.

### Sample collection and storage

A total of 100 clinical isolates of *Candida* species were collected from Khartoum state hospitals, Sudan. The isolates were pre-identified at each hospital’s laboratory using conventional methods such as wet mount, gram stain, germ tube, and growth on Sabouraud Dextrose Agar media (SDA). Immediately after collection, the isolates were grown into SDA (M063, HIMEDIA, Mumbai, India) at 32°C for 24–48 hours. From the grown culture, colonies were picked and streaked over a slant of SDA in screw-cap tubes, each slant was filled with sterile liquid glycerol and tightly closed and stored in 4 C° until recovered.

### Identification

Chromogenic media Hi-Chrome *Candida* differential agar media supplemented with chloramphenicol 0.5g/L (M1297A, HIMEDIA, Mumbai, India) was used to differentiate between *Candida* species based on colonies’ color and morphology. A subculture from the stock culture was allowed to grow on SDA for 24 hour, subsequently one well isolated colony from the grown culture was picked out and streaked over the prepared Hi-Chrome media and incubated at 32°C for 24–48 hours, the result was interpreted as per manufacture instructions ( *C. albicans*—light green, *C. glabrata*—cream to white, *Candida krusei*—pale, fuzzy and *C. tropicalis*—blue to purple, *C. dupliensis*—pale green) ^25^.

### Antimicrobial sensitivity testing

Sensitivity testing to all isolated *Candida* species was carried out as recommended by the Clinical Laboratories and Standard Institute (CLSI) ^26^. A modified Mueller Hinton Agar media (M173, HIMEDIA, Mumbai, India) supplemented with 2% glucose and methyl blue 5µg/mL was used. Using overnight culture on SDA, the inoculum was prepared by suspending 4 well-isolated colonies in 5mL sterile saline, inoculum size was adjusted by matching the turbidity with standard McFarland which was prepared by adding 0.5 mL BaCl2 (0.048 mol/L) to 99.5 mL H _2_SO _4_. Within 15 minutes after adjusting the turbidity and by using sterile cotton swab, the microorganisms were streaked from the center of the petri dish to the top, each time the plate was rotated 60° to ensure that the agar surface is at least double streaked. Within 15 minutes after streaking, three discs, namely fluconazole 25µg, itraconazole 10µg and amphotericin 10µg (SD232, SD221, SD111. HIMEDIA, Mumbai, India) were applied to each inoculated petri dish, gently pressed into the agar using sterile forceps, incubated at 32C° for 24–48 hours, zone dimeter around each disc were measured using calipers and result was interpreted as per CLSI ^26^.

### Genomic DNA extraction, gene amplification, detection and sequencing

Genomic DNA was extracted using guanidine chloride method, briefly, DNA extraction was carried out using 48 hours grown culture on SDA media, three to five colonies were washed with 5 mL phosphate buffer saline (PBS) three times, then 2 mL white cell lysis buffer and 20 μL of proteinase K (10 mg/mL; iNtRON Inc, Korea) were added to the pellet in a Falcon tube, vortexed and incubated at 37°C overnight. Then 1 mL from guanidine chloride (7M; iNtRON Inc, Korea) and 350 μL of ammonium acetate (7M; Loba Chemie, India) were added. The tubes were vortexed and incubated at 65°C in a water bath for 2 hours. After that 2 mL pre chilled chloroform (sd Fine-Chem limited, India) was added, and centrifuged at 6000 RPM for 20 minutes and the supernatant was transferred into a new Falcon tube and completed to 10 mL volume with pre chilled absolute ethanol (Carlo Erba, France) and incubated overnight at -20°C for completion of DNA precipitation. After incubation the tubes were centrifuged at 6000 RPM for 20 minutes, then the ethanol was poured off and the same step was repeated with 70% ethanol. After that the tubes were left to air dry. Finally, DNA was suspended in 80 μL TE buffer (iNtRON Inc, Korea) and incubated at 4 °C until used ^27^, as a template for PCR. The *ERG11* gene from *C. albicans* was amplified using previously published primers ^14, 28^: forward: (5′-CAAGAAGATCATAACTCAAT-3′) and reverse (3′-AGAACACTGAATCGAAAG-5′) (Macrogen Inc. Korea). All PCRs were carried out in final volume of 20 µL containing *Maxime* PCR PreMix Kit *i*-Tag (2.5U *i*- *Tag*TM DNA polymerase, 2.5mM each dNTPs, 1x reaction buffer and 1x Gel loading buffer), 1µL each forward and reverse primer (10pmol final concentration), 2.5µL genomic DNA, and the volume was completed with distilled water. The PCR was carried out in G-storm thermocycler with the following conditions: initial denaturation at 94°C for 4 min; 35 cycle of denaturation at 95°C for 30 s, primer annealing at 55°C for 30 s, and extension at 70°C for one min; followed by final extension step at 72°C for 10 min. The final product was visualized by loading 3µL in 0.8% agarose gel electrophoresis for 45 minutes under the voltage 100 V. Distinct bands were observed under UV light and photographed. The *ERG11* gene from the most *C. albicans* resistant isolates (isolate 10, 13, and 14) and one sensitive isolate (24 randomly selected, double blinded by independent researcher) were selected for sequencing (Sanger sequencing in BGI, Shenzhen, China). All sequences were deposited in GenBank and accession numbers MT081007, MT081008, MT081009, MT081010 for isolate 10, 13, 14, and 24, respectively, were obtained. Sequences were aligned based on fluconazole susceptible strain SC5314 (GenBank accession number X13296) using BioEdit software version 7.2.5.

## Results

### Collection and identifications

Out of 100 samples collected, 80 were from females and 20 from males with a mean age of 40. 66 isolates were from urine samples, 17 from sputum, 12 were high from vaginal swabs, and 5 from other sites. Overall, the most common species was *C. albicans* (n=51), while the most prevalent Non- *albicans* species was *C. glabrata* (n=31), followed by *C. krusei* (n=8). Table 1 provides a detailed description on the frequency of each species with respect to the site of isolation ^29^.

**Table 1.** Different site of isolations in relation to frequency of each *Candida* species identified.

	*Candida* species						
Specimens	*C. albicans*	*C. glabrata*	*C. kruise*	*C. tropicalis*	*C. dupliensis*	Total	Overall rate of resistance
Urine	34	20	6	3	3	66	(19) 28.78%
Sputum	7	7	0	1	2	17	(3) 17.64%
Vaginal swab	9	1	1	1	0	12	(3) 25%
Throat swab	1	2	0	0	0	3	(0) 0%
Blood	0	0	1	0	0	1	(1) 100%
Catheter	1	0	0	0	0	1	(0) 0%
Total	51	31	8	5	5	100	(26) 26%

### Antifungal sensitivity testing (AST)

Fluconazole was the least effective agent followed by itraconazole. Itraconazole resistance was observed among Non- *C. albicans* (NCA) species, high frequency of intermediate susceptibility dose-dependent (ISDD) was observed for itraconazole among all *Candida* species, while there was no amphotericin resistant isolate detected. Rate of resistance among female isolates 28.75% (23 out of 80) was higher than the male 15% (3 out of 20).

Fluconazole resistance was observed in 23% of *C. albicans* samples, only 2 isolates were ISDD, and the remaining isolates (72.5%) were sensitive, there were no itraconazole and amphotericin resistant *C. albicans* isolates. However, 31% were categorized as ISDD to itraconazole. Among NCA species, 19.4% of *C. glabrata* were fluconazole resistant, as well as all *C. krusei* isolates (8). Azole cross resistance was observed among 2 *C. glabrata* and 2 *C. krusei* isolates. One *C. dupliensis* was resistant to fluconazole while there were no *C. tropicalis* resistant isolates. Complete AST results are shown in Table 2 ^29^.

**Table 2.** The sensitivities of different species towards the selected antifungal drugs.

Species	Drug	Category			Total
		Sensitive (%)	Intermediate (%)	Resistant (%)	
*C. albicans*	F I A	37 (72.5%) 35 (68.6%) 49 (96%)	2 (4%) 16 (31.4%) 2 (4%)	12 (23.5%) 0% 0%	51 51 51
*C. glabrata*	F I A	25 (80.6%) 7 (22.5%) 15 (48.3%)	0% 22 (71%) 16 (51.7%)	6 (19.4%) 2 (6.5%) 0%	31 31 31
*C. krusise*	F I A	0% 4 (50%) 1 (12.5%)	0% 2 (25%) 7 (87.5%)	8 (100%) 2 (25%) 0%	8 8 8
*C. tropicals*	F I A	3 (60%) 4 (80%) 5 (100%)	2 (40%) 1 (20%) 0%	0% 0% 0%	5 5 5
*C. duplinienis*	F I A	4 (80%) 2 (40%) 5 (100%)	0% 3 (60%) 0%	1 (20%) 0% 0%	5 5 5

F: Fluconazole, I: Itraconazole, A: Amphotericin.

### *C. albicans ERG11’s* gene amplification, electrophoresis analysis and sequencing

**Figure 1.**
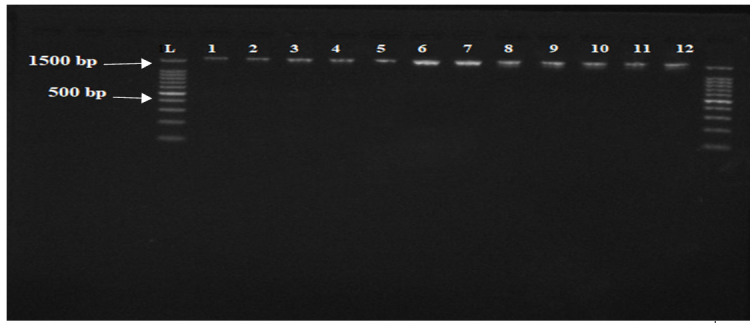
Agarose gel Electrophoresis showing the bands of amplified *ERG11* gene. From left to right lanes: L, 100 bp DNA ladder, 1 to 12 lanes are the amplified *Candida albicans ERG11*’s gene (1587 bp).

The complete *ERG11* gene coding region (1587 bp) from *C. albicans* resistant isolates (12) and one sensitive isolate (control) were amplified as shown in Figure 1 ^30^. Sequence analysis revealed 15 different mutations, 12 of which were silent, and 3 were non-synonyms Table 3. T495A and G1609A were observed only in resistant isolates (isolate 10 and 14 respectively), while A945C was observed in sensitive and resistant isolates (isolate 13, 14, and 24). All mutations were previously reported ( Table 3 ^31^).

**Table 3.** Different mutations of ERG11 gene and amino acid substitutions.

Isolates number	Position of mutation	Mutation From - To	Amino acid substitution
Silent mutation (s)			
10, 13, 14, and 24	462	T – C	No
13, 14, and 24	558	C – T	No
13 and 14	696	T – C	No
10, 13, 14, and 24	805	C – T	No
10, 13, 14, and 24	1143	T – C	No
10	1167	A – G	No
13, 14, and 24	1173	A – G	No
10, 13, 14, and 24	1257	C – T	No
13, 14, and 24	1350	T – C	No
13, 14, and 24	1443	C – T	No
10, 13, 14, and 24	1449	T – C	No
10	1587	A – G	No
**Non- synonym mutation (s)**			
10	495	T – A	*Asp 116 Glu*
13, 14, and 24	945	A – C	*Glu 266 Asp*
14	1609	G – A	*Val 488 Ile*

Asp= Aspartic acid, Glu= Glutamic acid, Val= Valine, Ile= Isoleucine All sequences were aligned based on *C. albicans* reference strain, with X13296 GenBank accession number.

## Discussion

In the current study, 100 clinical isolates of different *Candida* species were collected, identified, and their susceptibilities to fluconazole, itraconazole, and amphotericin were determined. The *ERG11* gene was amplified from some resistant isolates to investigate the impact of different mutations in the enzyme activity and hence drug susceptibility.

Nearly half of the samples collected were *C. albicans* (51%), while *C. glabrata* (31%) and *C. krusie* (8%) being the most prevalent of the other species identified ( Table 1 ^29^). *C. albicans* remains to be the most common species (51%), a similar finding was obtained in Sudan, in 2008, in a study that aimed to characterize vaginal candidiasis among pregnant woman indicating that the prevalence was 81% ^32^. More recently in 2018 a study conducted on cancer patients at the Isotope and Radiation Centar in Sudan, concluded that the prevalence of *C. albicans* was 59% ^33^, however, these numbers indicate that the prevalence of non- *Candida albicans* species are increasing: 19% in 2008, 41% in 2018, and 49% in this study. This result indicates the necessity of culturing any suspected *Candida* infections at species level for proper management.

Antimicrobial sensitivity testing reveals that fluconazole was the least effective agent, followed by itraconazole, while there were no amphotericin resistant isolates ( Table 2 ^29, 30^). Fluconazole resistance rate was 23%, this finding strictly similar to the finding from Nigeria 24% and India 20%, 19.5% ^34– 36^. A higher rate of resistance was reported in Ethiopia 38% and more recently 2020 in Ghana (48.1%) ^37, 38^. On the other hands, a lower resistance rate was reported in Egypt 11% ^39^. Regarding Itraconazole resistance, as few as 4 isolates of *C. krusie* and *C. glabrata* were resistant, this is in contrast to the finding from Ethiopia and Burkina Faso in which itraconazole resistance was as higher as 50 and 52% respectively ^37, 40^. This variation in susceptibility rate might be attributed to difference in previous exposure to fluconazole since several studies have described that azole resistance is highly associated with previous exposure to fluconazole ^40, 41^. Concerning azole cross-resistance, all isolates that were itraconazole resistant were fluconazole resistance, similar finding was obtained previously in which the cross-resistance rate was 83%. Unlike azoles, amphotericin was the most effective agents (100%) a comparable finding was obtained in Egypt (98.7%), Ethiopia (93.7%), India (90%), and Ghana (87.2%) ^35, 37– 39^. This potent susceptibility profile of amphotericin could be due to it is limited uses.

It has been observed that drug resistant fungal pathogens are increasing, and reduced susceptibility to azoles, especially fluconazole, along with azole cross resistance was detected ^3^. According to these findings we believe that there is urgent need for AST, especially when physicians intend to prescribe fluconazole as it is the least effective agents, or in such settings that non *- C. albicans* species are suspected as they possess relatively high rates of resistance.

The *ERG11* gene was sequenced from some *C. albicans* resistant isolates and one sensitive isolate for the purpose of examining the impact of different mutations (if present) on fluconazole resistance. The three detected mutations (T495A, A945C, and G1609A, which precipitate D116E, E266D, and V488I aa substitutions respectively) have been described previously in both sensitive and resistant isolates, and strongly suggesting that they are not contributed directly to resistance.

In the present study, E266D aa substitution which was described by some authors as the most common polymorphism in the *ERG11* gene has been detected in both sensitive (isolate 24) and resistant (isolate 13, 14) isolates ( Table 3 ^31^), so our finding completely agree with previous data which concluded that this mutation alone has no role in resistance ^28, 42^.

In our analysis, D116E and V488I aa substitutions were detected only in resistant isolates (isolate 10 and 14 respectively, Table 3 ^31^); the same results have been described previously ^28, 43^; however, the detection of these mutation in fluconazole susceptible isolates indicates that they lack a vital role in the development of resistance ^14^. In isolate 14, E266D and V488I were found together, a similar finding was obtained previously ^43^. The impact of E266D occurring simultaneously with other mutations such as K143R, F145L, and G464S have been well characterized using site directed mutagenesis ^42^, unlike the coexistence of E266D and V488I which is needed to be more clarified.

One of the limitation in this study is that we are unable to detect some regions and therefore some mutations at the beginning and/or end of the *ERG11* gene because of their low quality (common problem in Sanger sequencing for sequences more than 1000 bp) ^44^. We have tried to solve this problem by using either forward or reverse sequencing reads, however, it was very difficult to double check some of these mutations for further confirmations. Our recommendation in this regard is to consider different sequencing techniques that are able to detect the entire region (1587bp) reliably. 

According to our results, *ERG11* gene mutations in *C. albicans* was not the main causes of resistance, our future recommendations lay on considering alternative resistance mechanisms, more especially, studying the expression level of *CDR1, CDR2, MDR1,* and *ERG11* genes which expected to give a complete view of the resistance processes.

## Conclusion

Nearly half of the identified isolates were C. *albicans*, and the most prevalent non- *C. albicans* was *C. glabrata*. Among all antifungals tested, fluconazole was the least effective, while all isolates were sensitive to amphotericin. The detected missense mutations were not directly associated with fluconazole resistance; however, resistance among these isolates might be due to other mechanisms such as efflux pump gene overexpression.

## Data availability

### Underlying data

All sequences were deposited in GenBank under accession numbers MT081007, MT081008, MT081009, and MT081010 for isolate 10, 13, 14, and 24, respectively.

Figshare: demographic, identification, sensitivity test data. https://doi.org/10.6084/m9.figshare.12449615.v1 ^29^

This project contains the following underlying data:

- sample collection sheet for F1000.xlsx (A spreadsheet contain data regarding patients age, gender and site of isolation, species assay results for each sample coupled with susceptibility to fluconazole, itraconazole and amphotericin).

Figshare: sequence data for ERG11 gene. https://doi.org/10.6084/m9.figshare.12600128.v1 ^31^

This project contains the following underlying data:

- 10__[19070622] F__D01_1907004923G.ab1 (Raw sequence data for opening in Finish TV for isolate 10, forward sequencing).
- 10__[19070624]R__A02_1907004923G.ab1 (Raw sequence data for opening in Finish TV for isolate 10, reverse sequencing).
- 13__[19070622]F__E01_1907004924G.ab1 (Raw sequence data for opening in Finish TV for isolate 13, forward sequencing).
- 13__[19070624]R__B02_1907004924G.ab1 (Raw sequence data for opening in Finish TV for isolate 13, reverse sequencing).
- 14__[19070622]F__F01_1907004925G.ab1 (Raw sequence data for opening in Finish TV for isolate 14, forward sequencing).
- 14__[19070624]R__C02_1907004925G.ab1 (Raw sequence data for opening in Finish TV for isolate 14, reverse sequencing).
- 24__[19070622]F__G01_1907004926G.ab1 (Raw sequence data for opening in Finish TV for isolate 24, forward sequencing).
- 24__[19070622]F__G01_1907004926G.ab1 (Raw sequence data for opening in Finish TV for isolate 24, reverse sequencing).

Figshare: candida identification using Hi-chrome media and gel electrophoresis for ERG11 gene. https://doi.org/10.6084/m9.figshare.12775769.v1 ^30^

This project contains the following underlying data:

- ERG11 gene from C. albicans.jpg (Raw image for PCR gel for ERG11 gene).
- 131733_2018.3.7.jpg (Raw images for identification of isolates 1-9, inverted plate).
- 131739_2018.3.7.jpg (Raw images for identification of isolates 1-9, upright plate).
- 131718_2018.3.7.jpg (Raw images for identification of isolates 15-22, inverted plate).
- 131724_2018.3.7.jpg (Raw images for identification of isolates 15-22, upright plate).
- 131630_2018.3.7.jpg (Raw images for identification of isolates 27-34, upright plate).
- 131637_2018.3.7.jpg (Raw images for identification of isolates 27-34, inverted plate).
- 131532_2018.3.7.jpg (Raw images for identification of isolates 45-52, inverted plate).
- 131541_2018.3.7.jpg (Raw images for identification of isolates 45-52, upright plate).
- 131555_2018.3.7.jpg (Raw images for identification of isolates 56-63, upright plate).
- 131605_2018.3.7.jpg (Raw images for identification of isolates 56-63, inverted plate).
- 131619_2018.3.7.jpg (Raw images for identification of isolates 64-71, upright plate).
- 131645_2018.3.7.jpg (Raw images for identification of isolates 64-71, inverted plate).
- 131659_2018.3.7.jpg (Raw images for identification of isolates 73-80, inverted plate).
- 131708_2018.3.7.jpg (Raw images for identification of isolates 73-80, upright plate).
- 131508_2018.3.7.jpg (Raw images for identification of isolates 82-86, upright plate).

Data are available under the terms of the Creative Commons Attribution 4.0 International license (CC-BY 4.0).
