# Supplementary material for: Detection of antifungal drug-resistant and ERG11 gene mutations among clinical isolates of Candida species isolated from Khartoum, Sudan
Source: F1000Res. 2021 Jan 29;9:1050. Originally published 2020 Aug 26. [Version 2] doi: 10.12688/f1000research.24854.2 (PMC8114185; doi:10.12688/f1000research.24854.2)
Supplement: Peer review history

# Peer review: Reviewer response for version 2

**DOI:** 10.5256/f1000research.54119.r84350

**Published:** 2021-05-11

**Authors:** Muhammad Sajid Hamid Akash

**Affiliations:** 1 Department of Pharmaceutical Chemistry, Government College University, Faisalabad, Pakistan

1) No need for subtitle in the introduction.

 2) It will be better if the author could provide more resistance reports for his region in the introduction. Instead of extensively describing unnecessary parts such as the efflux pump as it is not covered in the study methodology. 

 3) The most important finding must be stated in the first most paragraph of the discussion.

Is the work clearly and accurately presented and does it cite the current literature?

Yes

If applicable, is the statistical analysis and its interpretation appropriate?

Yes

Are all the source data underlying the results available to ensure full reproducibility?

Yes

Is the study design appropriate and is the work technically sound?

Yes

Are the conclusions drawn adequately supported by the results?

Partly

Are sufficient details of methods and analysis provided to allow replication by others?

Yes

Reviewer Expertise:

Pharmaceutical Analysis

I confirm that I have read this submission and believe that I have an appropriate level of expertise to confirm that it is of an acceptable scientific standard.

# Peer review: Reviewer response for version 1

**DOI:** 10.5256/f1000research.27422.r73504

**Published:** 2020-11-11

**Authors:** Raja Ahsan Aftab

**Affiliations:** 1 Faculty of Health and Medical Sciences, School of Pharmacy, Taylor’s University, Subang Jaya, Malaysia

The study is about anti-fungal sensitivity testing and detection of resistant gene/s. It is important to carry out such kinds of studies to help prescribers and policymakers in making better future plans and guidelines for the treatment of candida infection.

- It would be better if the authors could clarify why these 3 anti-fungal drugs were selected for the sensitivity testing in the introduction.
- The authors need to add a few more studies and comparisons about the candida resistance during the discussion.
- The authors collected 100 samples from different sites and genders, the exact percentage of resistance/sensitive isolates with regard to gender and site of isolation can be added to the tables or as a text.

Is the work clearly and accurately presented and does it cite the current literature?

Yes

If applicable, is the statistical analysis and its interpretation appropriate?

Not applicable

Are all the source data underlying the results available to ensure full reproducibility?

Yes

Is the study design appropriate and is the work technically sound?

Yes

Are the conclusions drawn adequately supported by the results?

Yes

Are sufficient details of methods and analysis provided to allow replication by others?

Yes

Reviewer Expertise:

Clinical Pharmacy , infectious disease , nephrology , treatment outcomes

I confirm that I have read this submission and believe that I have an appropriate level of expertise to confirm that it is of an acceptable scientific standard.

# Response

**Published:** 2021-01-25

**Authors:** Ahmed Kunna

**Affiliations:** Sudan International University, Sudan

Dear Prof Raja Ahsan Aftab:

 Thank you for your valuable comments.

 In response to your 3 comments we have:

1. Justified the reasons for selecting the 3 antifungals in the Introduction.
2. We have added the percentage of resistant isolates in relation to gender in Result section, and the percentage of each site of isolations as crosstabulation in Table 1.
3. We have comprehensively discussed the pattern of antifungal resistance especially in our region (Africa) in the Discussion section by adding 7 additional citations.

# Peer review: Reviewer response for version 1

**DOI:** 10.5256/f1000research.27422.r70299

**Published:** 2020-09-07

**Authors:** Rasoul Mohammadi

**Affiliations:** 1 Department of Medical Parasitology and Mycology, School of Medicine, Isfahan University of Medical Sciences, Isfahan, Iran

This manuscript is a common epidemiological study. It has some major drawbacks including:

1. Isolates were identified by phenotypic methods. Genus of *Candida *contains about 200 species and identification needs to confirm by molecular techniques. Conventional methods are not enough to identify *Candida *spp.
2. The majority of isolates were obtained from urine and sputum (66+17). *Candida *spp. are normal flora and authors have to confirm the infections caused by these fungi. 

Isolation of *Candida *from urine (candiduria) is not an infection, we have some criteria for urinary tract infections (UTIs), and the authors have to confirm it.

 Isolation of *Candida *from sputum is not important because we can isolate *Candida *spp. from sputum, even in immunocompetent individuals.

Is the work clearly and accurately presented and does it cite the current literature?

Partly

If applicable, is the statistical analysis and its interpretation appropriate?

No

Are all the source data underlying the results available to ensure full reproducibility?

Yes

Is the study design appropriate and is the work technically sound?

No

Are the conclusions drawn adequately supported by the results?

Yes

Are sufficient details of methods and analysis provided to allow replication by others?

Yes

Reviewer Expertise:

Medical Mycology

I confirm that I have read this submission and believe that I have an appropriate level of expertise to state that I do not consider it to be of an acceptable scientific standard, for reasons outlined above.

# Response

**Published:** 2021-01-27

**Authors:** Ahmed Kunna

**Affiliations:** Sudan International University, Sudan

Dear Prof Rasoul Mohammadi;

 Thank you for comprehensively reviewing our paper and for your appreciated comments:

 Regarding the first comments;

 The primary objective of the study was to isolate the medically important Candida species, characterizing Candida at species level is beyond the scope of the study. The sensitivity and specificity of Chromogenic media in differentiating as well as isolating common species such as ( *C. albicans*, *C. glabrata*, *C. Krusie*, and *C. tropicals*) is well documented in the previous studies (see *Evaluation of chromogenic media and seminested PCR in the identification of Candida species* ), according to previous publication in Sudan; more than 90% of infections due to Candida species was caused by this species (see *Internal transcribed spacer for identification of yeast species isolated from cancer patients at the Isotope and Radiation Center, Khartoum, Sudan: A cross-sectional, case-control study*). On the other hand, culturing was the efficient and the easiest way to isolate the mixed isolates within the same sample.

 With respect to the second comments;

 Candiduria is considered one of the most controversial issues in patient management. As well as isolation of Candida from oral cavity since this species is a commensal microbe. During the course of sample collections (1 year), we have tried to overcome this problem by carefully selecting our isolates for eligibility since:

1. All of isolates were derived from a patient with some risk factors such as *Diabetes* mellitus, hospitalised patient, and elderly patients.
2. All isolates were recovered from patients with characteristic sign and symptoms for UTI, while the growth of bacteria was insufficient to prove bacterial infections. In most of the cases urine analysis as well as culture were repeated.  
3. The final decision of considering Candida as a sole causative agent was made by a physician, upon that recommendations sample were considered eligible.
